# Design of Pyrrole-Based Gate-Controlled Molecular Junctions Optimized for Single-Molecule Aflatoxin B1 Detection

**DOI:** 10.3390/s23031687

**Published:** 2023-02-03

**Authors:** Fabrizio Mo, Chiara Elfi Spano, Yuri Ardesi, Massimo Ruo Roch, Gianluca Piccinini, Mariagrazia Graziano

**Affiliations:** 1 Department of Electronics and Telecommunication, Politecnico di Torino, 10129 Torino, Italy; 2 Department of Applied Science and Technology, Politecnico di Torino, 10129 Torino, Italy

**Keywords:** AFB1, aflatoxin, amperometric detection, atomistic simulations, electrical detection, gold electrodes, molecular FET, molecular junction, single-molecule electronics, single-molecule sensor, single-molecule FET, pyrrole, 8PyDT

## Abstract

Food contamination by aflatoxins is an urgent global issue due to its high level of toxicity and the difficulties in limiting the diffusion. Unfortunately, current detection techniques, which mainly use biosensing, prevent the pervasive monitoring of aflatoxins throughout the agri-food chain. In this work, we investigate, through ab initio atomistic calculations, a pyrrole-based Molecular Field Effect Transistor (MolFET) as a single-molecule sensor for the amperometric detection of aflatoxins. In particular, we theoretically explain the gate-tuned current modulation from a chemical–physical perspective, and we support our insights through simulations. In addition, this work demonstrates that, for the case under consideration, the use of a suitable gate voltage permits a considerable enhancement in the sensor performance. The gating effect raises the current modulation due to aflatoxin from 100% to more than $10^{3}÷10^{4}$%. In particular, the current is diminished by two orders of magnitude from the μA range to the nA range due to the presence of aflatoxin B1. Our work motivates future research efforts in miniaturized FET electrical detection for future pervasive electrical measurement of aflatoxins.

## 1. Introduction

Aflatoxins are dangerous low weight mycotoxins mainly produced as secondary metabolites of the *Aspergillus flavus* and *Aspergillus parasiticus*, which are present in various parts of the extracellular matrix constituting the fungi biofilm [1,2,3]. Six aflatoxins have been identified, known as aflatoxin B1 (AFB1), with corresponding metabolite AFM1; aflatoxin B2 (AFB2), with corresponding metabolite AFM2; aflatoxin G1 (AFG1); and aflatoxin G2 (AFG2). Among them, AFB1 is the most diffused and dangerous; it belongs to Group I of carcinogens to humans and causes several health consequences such as thymic aplasia, liver and kidney pathologies, and chronic infections [3,4]. AFB1 is present in all steps of the agri-food chain, i.e., in the field, during harvest, in post-harvest storage, and even in successive steps. Mycotoxin enters the food chain by contaminating nuts, corn, rice, and other cereals, causing around 25% of the world’s harvest to be destroyed every year [2,3]. Aflatoxins can also enter the food chain through indirect processes, e.g., through contaminated milk produced by animals fed with contaminated fodder.

Pervasive aflatoxin concentration monitoring is still a distant objective due to the intrinsic limits of the state-of-the-art measuring techniques [1,5]. Current measuring methods generally rely on appropriate extraction and clean-up methods (e.g., liquid-liquid extraction, liquid-solid extraction, turbulent flow columns), which represent 66% of the entire measurement time and can significantly affect result precision [1,5]. Subsequent separation techniques such as High/Ultra-High Performance Liquid Chromatography (HPLC/UHPLC) or Capillary Electrophoresis (CE) should be used to make the aflatoxin available for measurement. After the separation, an Enzyme-Linked Immunosorbent Assay (ELISA) can be used for rapid and quantitative detection of the molecule [5]. Even if these detection methods show high sensitivity and are excellent for screening, they require bulky equipment, extended test times, and skilled lab technicians [1,2,5], thus motivating researchers to seek alternative methods. For example, lateral flow devices are simple and cheap, although they are still far from enabling pervasive monitoring, since they are limited by precision and disposability [6]. Moreover, quantitative measurements are possible exclusively in liquids, even if aflatoxins are naturally present in a solid or gas matrix. Therefore, only a few samples of the total amount of food undergo extraction and testing. The capability of measuring the aflatoxin concentration in solid/gas matrices is crucial in the biological research scenarios and would assure large scale monitoring [5]. Recently, nanotechnology has been demonstrated to be effective in developing novel aflatoxin detection techniques, e.g., through nanoparticle-based assays [7]. Furthermore, in the literature, there are increasing works concerning on-site detection devices, such as portable electrochemical and bio-sensing devices for rapid on-site detection of pesticides [8,9]. The possibility of integration of smartphone technology with both portable electrochemical sensing platforms [8,9] and nanotechnology is also reported, for example, the use of a smartphone-based quantitative device exploiting gold nanoparticles (GNPs) and time-resolved fluorescence microspheres (TRFMs) for multiplex mycotoxins detection [10]. Nanosize detection elements show an intrinsically high sensitivity and resolution, which imply accurate quantitative detection [7]. For example, surface-enhanced Raman scattering (SERS)-based immunoassays, with silica-encapsulated hollow gold nanoparticles (SEHGNs), demonstrate high sensitivity to AFB1 [11].

In this scenario, the recent developments in nanotechnology and nano-fabrication techniques have made single-molecule electrical detection possible through a variety of nanodevices such as nanopores, nanogaps or nanopipette devices [12,13,14]. Electrical measurements are possible through a suitable probe, i.e., a selective detection element, or by direct measurement of the analyte conductance. Even if noise strongly affects the nanoscale experimental setups, sensor parallelization and artificial intelligence techniques appear promising to overcome this issue [14]. Furthermore, the sensing community is currently attracted by molecular electronics, which implements electronic components through single molecules or small molecular ensembles [15,16,17]. Scanning Tunneling Break Junction (STM-BJ) techniques and Mechanical Break Junction (MBJ) platforms allow the experimental verification of single molecule devices such as switches, photoswitches, diodes, memristors and sensors [18,19,20,21,22]. Molecular electrical detection has been demonstrated in various applications experimentally and theoretically [22,23,24,25,26], and significantly novel properties have been highlighted by the use of molecules in the realization of sensors [25]. In addition, possible nanoscale phenomena with exclusive conductive properties, e.g., the quantum interference [27,28,29], and successful applications in high-sensitivity sensors make it very promising for the detection of small molecules.

Recently, we investigated in a previous work [26] gold-8-pyrrole-dithiol molecular dots (Au-8PyDT) as amperometric Single-Molecule Sensors (SMS) for AFB1 detection, which resulted in encouraging results for employing molecular electronics for detection. In particular, a direct sensing principle, not relying on the presence of bio-transducers, makes the molecular sensor reusable, thus promising for real-time, on-site, label-free, pervasive automatic detection of AFB1 [26]. Furthermore, the use of a single poly-pyrrole polymeric chain as a detection element promotes high miniaturization of the sensing element, which boosts the sensitivity, and paves the way for the mass monitoring of AFB1 in the agri-food chain from farm to fork. Finally, the poly-pyrrole has proven to be bio-compatible and non-toxic, and therefore overcomes the drawback of the toxicity of commonly used nanomaterials [30,31].

The present work provides a deeper insight into the physical–chemical features of Au-8PyDT molecular junctions. We explain the effect of different source and drain contact shapes on the dominant electronic transport mechanism, which enables the sensing of AFB1. Furthermore, we also address an enhanced device structure with a third gate terminal electrostatically coupled to the polymeric channel of the sensor. The proposed SMS constitutes a molecular quantum dot-based Field Effect Transistor (FET), or single-Molecule FET (MolFET), in which a suitable gate threshold voltage triggers electronic conduction. We then investigate the sensing performance of the proposed MolFET, demonstrating that the gate terminal can be an effective engineering tool to control and boost SMS sensitivity to AFB1, and we clarify the effects of the gain on the electrical detection of AFB1. We find the proposed molecular transistor SMS enhances AFB1 detection. Our simulation results and analysis motivate further research on quantum dot-based FETs as ultra-sensitive single-molecule detection elements in future pervasive sensory systems.

Finally, note that the proposed single-molecule sensor significantly differs from conventional macro- and micro-scale FETs based on conjugated polymers [32,33,34,35]. Indeed, the intrinsic nanoscale size of the 8PyDT-FET means that it is governed by ballistic quantum conduction, and it is not possible to define average quantities that usually characterize macroscale devices. For example, macroscopic polymers are often dominated by hopping electronic transport and interactions among the different polymeric chains, which results in statistical ensemble properties such as the definition of electron mobility, whereas statistical quantities are not well-defined in the electronic transport mechanisms of single-molecule ballistic devices due to the minor role of scattering processes occurring on this scale [36]. Despite the differences, the possibility to integrate amperometric molecular electronics sensors within conventional silicon-based integrated circuits has recently been proved, thus making the single-molecule electrical detection appealing for future sensing platforms [37].

We summarize the peculiar single-molecule FET working and sensing principles in Section 2, the methodology and computational methods in Section 3 and the results in Section 4. The conclusions and future work are discussed in Section 5.

## 2. Theoretical Background

Molecular electronics, or Moletronics, implements electronic components through single molecules or small molecular ensembles [15,17]. Two terminal molecular quantum dot junctions can be employed as molecular resistors and capacitors [17]. Furthermore, the possibility of synthesizing ad hoc molecules with different and peculiar transport features permits implementing promising molecular electronic components [16,17]. For instance, Negative Differential Resistance (NDR), which cannot be observed in current solid-state technology, can be found in [3,3]paraCyclophane (pCp)-based molecular junctions and can be exploited in designing MolFET-based circuits [29,38].

**Figure 1:**
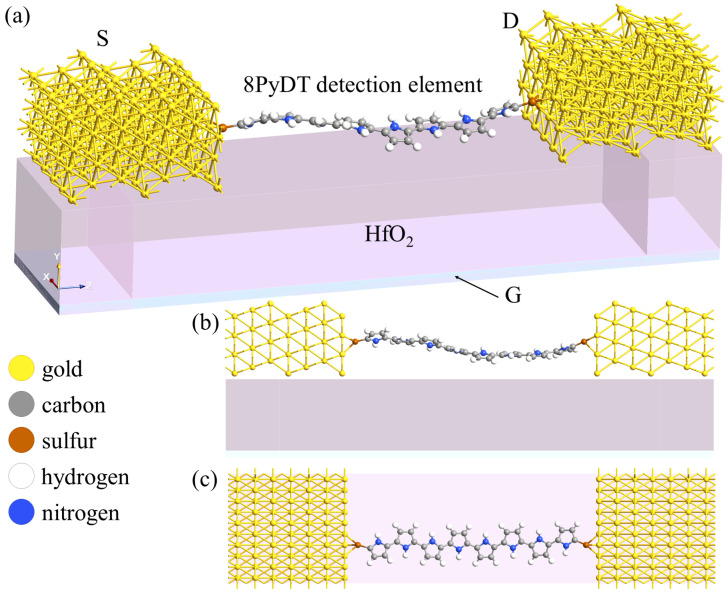
(**a**) 3D view of the structure of the proposed Au-8PyDT MolFET SMS; (**b**) side view; (**c**) top view.

Concerning two-terminal junctions, a third electrostatically coupled electrode is added to the molecular device to constitute a structure with three electrodes, namely source (S), drain (D) and gate (G) electrodes. The added electrode, i.e., the gate, permits modulating the conductive properties of the S–D junction, as we describe in detail in Section 2.2, to constitute a MolFET. Figure 1 depicts the MolFET we have studied in this work as an AFB1 sensor. We selected gold as the S and D electrode material since it offers good chemical properties, permitting the realization of molecular break junctions at room temperature [39,40]. In addition, gold is an inert metal, making it suitable for sensing applications, and gold nanogaps can be created by electromigration by crack-defined or mechanically controllable break junctions, thus also making the studied MolFET close to real experimental setups [41,42,43,44]. The detection element we chose was instead a single polymeric chain composed of a sequence of eight pyrrole monomers (8Py). Poly-pyrroles are known to present conductive properties similar to semiconductors and their sensing capabilities similar to both organic and inorganic polar and nonpolar analytes have already been proven [45]. Furthermore, the planarity of the poly-pyrrole and the fact that it mainly constitutes carbon atoms make it promising in terms of conduction properties at a single-molecule level. Indeed, molecular torsion and elements with different electronegativities can affect the orbital delocalization and strongly reduce the already small current by acting as electron localization centers. In addition, poly-pyrrole is non-toxic and biocompatible, contrary to commonly used nanomaterials [30,31]. In this work, we considered a poly-pyrrole chain constituted by eight monomers since its length perfectly matches the gold nanogap size accordingly to the supposed fabrication technique [46,47], thus easing the self-assembled fabrication process. The total nanogap size is 33.5 Å. Thanks to its extremely miniaturized size, only one AFB1 molecule at a time can interact with the 8Py sensing element because of steric hindrance repulsion, making the studied sensor an intrinsic single-molecule sensor. Since we selected gold as the electrode material, we propose to anchor the 8Py to the contacts through thiol anchoring groups (SH-), as typically done for organic molecules [15,16] and as experimentally demonstrated for pyrrole self-assembled monolayers on gold [48]. The 8Py molecule, together with the two thiols, constitutes the 8PyDT (8Py-Di-Thiol) molecular channel of the proposed SMS.

Finally, the proposed MolFET-based SMS comprises the gold-8PyDT-gold molecular junction (Au-8PyDT) and an additional G electrode. The G electrode is electrostatically coupled to the structure through a 10.67Å-thick HfO_2_ (relative dielectric permittivity 25), corresponding to two atomic layers (*c* direction, Baddeleyite structure in the monoclinic P2_1/c_ space group), achievable with Atomic Deposition Layer (ALD) fabrication processes [49,50].

### 2.1. Single-Molecule Junction as a Two-Terminal Device: Working Principles

This section considers the single-molecule two-terminal device composed of the 8PyDT molecular channel and the S and D electrodes. The three-terminal MolFET structure is analysed in Section 2.2, together with a description and the modelling of the G electrode.

**Figure 2:**
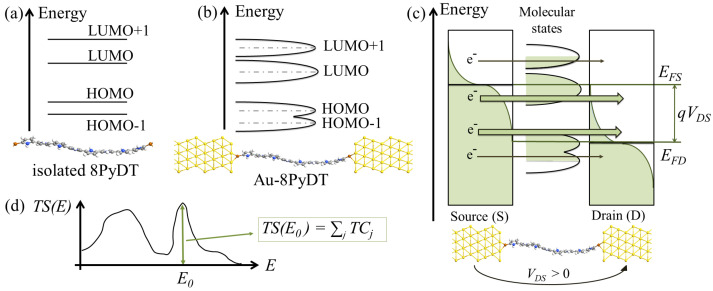
Pictorial representations of (**a**) discrete energy states of an isolated molecule (e.g., 8PyDT) and (**b**) broadened states of a molecular junction (e.g., Au-8PyDT junction). (**c**) Pictorial representation of the $V_{DS}$ effect in the Au-8PyDT. Transmission occurs mainly at energy *E* in between $E_{FD}$ and $E_{FS}$. Less transmission occurs at $E>E_{FS}$ and $E<E_{FD}$ due to the limited presence of occupied states in S and free states in D. (**d**) Pictorial representation of an example of the TS(E) function. At each fixed energy $E_{0}$, the $TS(E_{0})$ value is the superposition of the transmission probabilities (transmission eigenvalues) of the possible transmitting states (transmission eigenstates).

When the channel molecule, namely, the 8PyDT, is isolated, it exhibits its quantum mechanical nature through different discrete energy levels. Figure 2a shows the molecular energy levels close to the Fermi level, which are the most significant for transport, i.e., the Highest Occupied Molecular Orbital (HOMO), the HOMO-1, the Lowest Unoccupied Molecular Orbital (LUMO) and the LUMO+1. Thanks to the strong covalent bonds possible between gold and sulfur, the 8PyDT exhibits both a broadening and a shift in its energy levels when it is anchored to the S and D electrodes [51], see Figure 2b. Indeed, the Fermi level alignment occurring at thermodynamic equilibrium in the system composed of the 8PyDT and the S and D contacts produces a shift in the 8PyDT energy levels. Moreover, the chemical bond between thiol and gold contacts also facilitates electron movement between the 8PyDT and the S and D electrodes. Thus, electron states are no longer confined to steady states with infinite lifetimes. Instead, they are delocalized states with a finite lifetime, named intrinsic time (τ), comparable to the average time required to move an electron from/to the molecule to/from the contacts. The intrinsic time is related to the energy broadening (γ) through a time-energy uncertainty-like relation, τ≈h/γ, where *h* is Planck’s constant. Note that the lower the energy broadening, the larger the intrinsic time, i.e., the time the electrons spend in the molecular 8PyDT quantum dot on average. The limit case in which there is no uncertainty on the energy level value, i.e., γ→0, corresponds to infinite intrinsic time τ (no electron travel from/to the molecule to/from the contacts). γ→0 occurs when the molecule 8PyDT is isolated, i.e., electrons are in their quantum steady states for an infinite time with no possibility of escaping the system.

In the case of an applied (positive) D-S voltage, $V_{DS}$, the electron states that mainly contribute to conduction are those in between the D and S Fermi levels, $E_{FD}$ and $E_{FS}$, respectively. The energy range between the two Fermi levels is known as a bias window, defined as $BW=qV_{DS}$, where *q* is the electron charge. At zero kelvin, no S state is occupied above $E_{FS}$, meaning that no electron with an energy larger than $E_{FS}$ can leave the S and no free electron state is present below $E_{FD}$ in the drain, i.e., no electron can flow to D below $E_{FD}$. At higher temperatures, see Figure 2c, the occupation of electron states is mitigated by the Fermi–Dirac’s function. As a result, a few electron states with energy larger than $E_{FS}$ are occupied in S, and a few electron states with energy lower than $E_{FD}$ are free in D.

Considering the molecular device of interest in this work, its intrinsic nanometer size requires us to use a purely quantum mechanical treatment of the electron structure and transport. In addition, the sulfur–gold covalent bond guarantees a strong coupling regime between the molecular channel and contacts. Thus, τ is minimal, making the transport ballistic and minimizing the role of incoherent scattering [52,53]. In particular, we use the non-equilibrium Green’s function (NEGF) theory, since it is general enough to be functional in any operating condition, thus permitting a transport evaluation both in linear and non-linear regimes and in the case of high bias values [36]. Within the NEGF formalism, the current flow in the two-terminal device is calculated through Landauer’s formula for current [36,51]:

(1) $$I_{DS}=\frac{2q}{h}\int_{-\infty }^{+\infty }TS\left(E,V_{DS}\right)\left[f\left(E,E_{FS}\right)-f\left(E,E_{FD}\right)\right]dE$$

where $TS(E,V_{DS})$ is the so-called transmission spectrum, depending on both the electron energy (*E*) and the applied bias ($V_{DS}$). $f(E,E_{F})$ indicates the Fermi–Dirac distribution, with $E_{F}=E_{FS},\;E_{FD}$:

(2) $$f\left(E\right)=\frac{1}{e^{\frac{E-E_{F}}{kT}}+1}$$

where *k* is Boltzmann’s constant and *T* is temperature.

In the NEGF framework, the transmission spectrum, TS, is calculated with quantum mechanics. For the details, interested readers may refer to [36,51,54]. We here limit our attention to the most important equations and quantities required to understand the results section. In the following equation, all the quantum mechanical operators are intended in matrix form, e.g., $H_{M_{j,k}}=\int \psi_{j}^{*}{\hat{H}}_{M}\psi_{k}d\vec{r}$, with basis set ${\left\{\psi \right\}}_{m}$. The TS can be calculated from the Green’s functions as follows [51]:

(3) $$\begin{array}{cc} \; & TS=Tr\left[\Gamma_{S}G_{M}\Gamma_{D}G_{M}^{†}\right]\\ \; & with:\;\;\Gamma_{S,D}=i\left[\Sigma_{S,D}-\Sigma_{S,D}^{†}\right]\\ \; & and\;\;G_{M}={\left[\left(E+i\eta \right)S_{M}-H_{M}-\Sigma \right]}^{-1} \end{array}$$

where we dropped the *E* and $V_{DS}$ dependence to ease the notation. Tr is the trace operator, † indicates the complex conjugate and transpose, *i* the imaginary unit, $\Gamma_{S,D}$ are the S and D broadening functions, defined as the anti-Hermitian part of the relative contact self-energy $\Sigma_{S,D}$, $G_{M}$ is the molecule (or channel) Green’s function, η is a positive infinitesimal value repesenting the open boundary nature of the quantum system, $S_{M}$ is the channel overlap matrix, $H_{M}$ is the channel Hamiltonian operator and $\Sigma =\Sigma_{S}+\Sigma_{D}$.

Note that $G_{M}$ has the physical meaning of impulse response in the space and time domains for the considered molecular channel [51]. The self-energies, $\Sigma_{S,D}$, represent the effects of the electrode. In particular, the real part of $\Sigma_{S,D}$ (Hermitian) is related to the molecular level shift caused by the presence of the electrodes, whereas the anti-Hermitian part (i.e., $\Gamma_{S,D}$) is related to the molecular level broadening [51]. The trace operation returns the total transmission probability from S to D at the specific electron energy *E*, i.e., TS(E). It can be interpreted as the superposition of all the transmission coefficients (TCs) of the transmission states simultaneously contributing to the transport at *E*. Indeed, the electron transmission from S to D at energy *E* generally occurs through different spatial paths called Transmission Eigenstates (TEs). Each TE can be associated with a transmission probability TC. Notice that TS(E) can be larger than one since electrons populating the S at *E* can be transmitted to D through different TEs, each with a probability TC≤1. The TEs and the relative TCs are solutions of the quantum mechanical eigenvalue problem for the transmission operator, see Figure 2d.

The so-called charging effect is a notable effect that appears in nanoscale devices. An electron with an energy within the BW can fill an empty molecular channel energy level to contribute to the conduction. Nevertheless, the presence of this electron in the channel creates a repulsion for possible electrons entering the channel, and thus extra energy should be given to overcome the barrier caused by the repulsion of the first electron. In other words, the presence of the first electron raises the energy level of an amount equal to the extra energy that should be used to place the second one in the channel, and is named the charging energy [51]. Similarly, if two electrons initially occupy the energy level, the energy decreases when it becomes populated by only one electron. The charging effect is also present in macroscale devices, but it is negligible because of the large number of electron states in the channel. Indeed, the larger the number of free (and degenerate) energy states, the lower the energy to put another electron in the same energy level [51]. Consequently, the charging effect is usually not highly marked in a strong coupling regime between contacts and the molecular channel because of the wide broadening and more significant amount of (hybrid) states in the channel (with respect to the same channel in the weak coupling regime).

### 2.2. Single-Molecule FET: Working Principles

The transmission spectrum TS(E) of a single-molecule junction, and thus its resulting $I_{DS}$, is affected by several factors [55,56,57,58,59]: the class of molecule and its molecular conformation (i.e., chemical elements, bond orders, bond angles and lengths), the number of monomers (i.e., the molecular length), the linker functional and anchoring groups, the materials and the geometrical arrangements of the electrodes. In particular, the anchoring groups, the S/D electrode material and the geometrical arrangement influence the molecule–contact coupling strength ($\Gamma_{S/D}$) through the self-energies; thus, they strongly affect the broadening of the TS(E) peaks. By adding a third gate terminal, the single-molecule junction discussed previously becomes a single-molecule FET. The single-molecule FET, also called a MolFET, is the molecular counterpart of the conventional MOSFET. It is a three-terminal device in which the drain current, $I_{DS}$, is modulated by the gate voltage, $V_{GS}$. Usually, in MolFETs, the molecular channel is electrostatically coupled to solid-state gate electrodes [19,44,60,61], but electrochemical coupling also is possible with ionic liquid-gated systems [62,63]. Solid-state gate electrodes require additional nanofabrication steps when compared to two-terminal single-molecule devices. Furthermore, they poorly couple with the molecular channel because of the large distance between the molecular channel and the underlying gate stack due to the bulky gold S and D electrodes in between. Recently, the use of graphene electrodes has permitted better electrostatic gate coupling; thanks to its atomic thickness, the distance between the molecular channel and the underlying gate stack is considerably reduced [19]. Concerning electrochemical gate electrodes, additional nanofabrication steps are not required with respect to two-terminal single-molecule devices, and better gate coupling is achieved. Unfortunately, single-molecule electrochemical-gated devices raise the technological issues of stability and integration.

**Figure 3:**
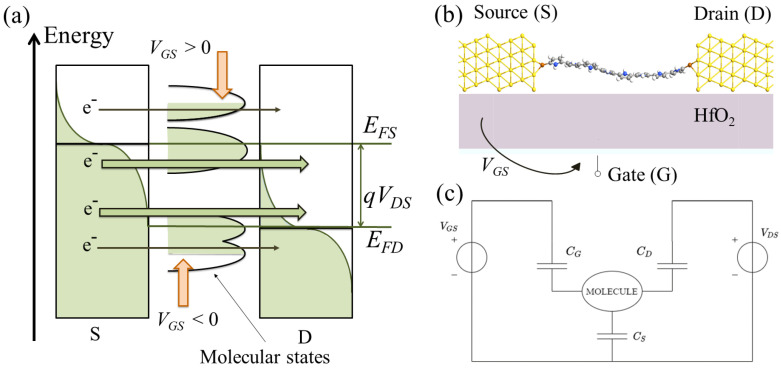
(**a**) Pictorial representation of the gating effect on a generic single-molecule FET; (**b**) representation of the structure of the proposed Au-8PyDT FET; (**c**) capacitive equivalent model of a generic MolFET.

In MolFETs with solid-state gate electrodes, the gate terminal defines the electrostatics of the system through the applied gate voltage, $V_{GS}$. The stack material, the spatial placement and the arrangement of the gate do not directly influence the chemical structure of the molecular junction nor the molecular channel transmission states contributing to conduction. Instead, they significantly determine the electrostatic control of the electrons flowing across the molecular channel, thus the effectiveness of the gate potential in the $I_{DS}$ current modulation during MolFET switching. The basic working principle of MolFET relies on the gating effect discussed in the following. The gating effect is the main effect of applying a gate voltage, $V_{GS}$, to the molecular junction. It consists of the upwards and downwards shift in the energy of the molecular energy states in the BW [51], as depicted in Figure 3a. This shift consequently causes a modulation of the current, $I_{DS}$, because of the entrance or exiting of molecular energy states that can contribute to electronic conduction. In particular, when a positive gate voltage ($V_{GS}$ > 0) is applied, the molecular states are shifted downwards in energy, whereas when a negative gate voltage is applied ($V_{GS}$ < 0), they are shifted upwards in energy. This gate-induced shift permits molecular states to enter or leave the BW, thus deciding their contribution to the electronic transmission. Quantitatively, the energy shift is due to the applied $V_{GS}$ and depends on the effectiveness of the gate potential energy, $U_{GS}$, on the electrostatic control of the channel molecular states. Using the capacitive equivalent model of the MolFET proposed in [51] and shown in Figure 3c, the gate effectiveness is defined through $U_{GS}$ as:

(4) $$U_{GS}=-q\frac{C_{G}}{C_{ES}}V_{GS}=-q\alpha V_{GS}$$

where $C_{ES}$ is the sum of all the capacitive contributions and $C_{G}$, $C_{D}$ and $C_{S}$, are the gate, drain and source capacitances, respectively. The ratio $C_{G}/C_{ES}$, often known in the literature as the gate coupling factor, α, quantifies the electrostatic coupling between the gate terminal and the molecular channel. The larger $C_{G}$ is, the closer α is to one, which implies a more significant energy shift of the channel molecular states, i.e., a significant electrostatic control performed by the gate on the modulation of current, $I_{DS}$. Maximum achievable values of gate coupling factor, α, in MolFETs with solid-state gate electrodes are around 0.3 [64,65,66]. In contrast, higher values can be achieved in single-molecule junctions with graphene-based electrodes and values are close to 1 in single-molecule devices with electrochemical gate electrodes [67]. Interestingly, by adequately tuning the gate voltage, $V_{GS}$, n-type (or p-type) devices can be realized by introducing the LUMO (or HOMO) molecular state into the BW. Furthermore, a proper engineering of the gate voltage may also enhance the ON/OFF current ratio in MolFETs, as shown in [29], and enhance the SMS sensitivity as we prove in Section 4.

### 2.3. Sensing Principle and Amperometric Detection

In a previous work [25], we have shown that chemical analytes can significantly modify the SMS transmission spectrum, TS(E), and thus the SMS current, $I_{DS}$, through a spatial modification of the region in which transmission occurs, i.e., by modifying the TEs contributing to conduction. In [25], we related the TE modification to an electron wavefunction spatial displacement caused by the presence of the analyte. In other words, the target analyte can significantly affect the electron wavefunction in the SMS by modifying the transmission properties TEs, TCs and TS(E), and therefore the current, $I_{DS}$, which can be evaluated through Equations (1) and (3). In addition, we have also shown in [26] that AFB1 can form hydrogen bonds with the 8PyDT detection element. A detailed treatment of hydrogen bonds can be found in [68]. For this work, it is enough to note that, similar to other bonds and non-bonding interactions, the hydrogen bond can affect the final geometrical displacement and orbitals of the interested chemical species. Therefore, the AFB1 presence significantly alters the wavefunction space distribution of the SMS by affecting its orbital conjugation. Consequently, it can significantly alter the TEs, TCs, TS(E) and $I_{DS}$.

**Figure 4:**
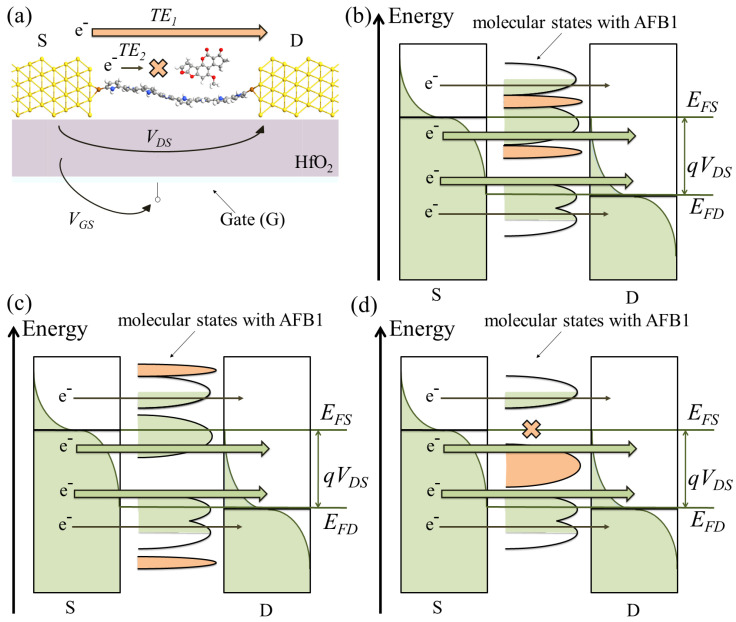
(**a**) Pictorial representation of the perturbation on the single-molecule FET caused by the target molecule (e.g., AFB1). The transmission, thus TEs, is altered. In this example, the presence of the target molecule enhances the transmission through TE1 and decreases the transmission through the TE2. Pictorial representations of the cases in which AFB1 significantly affects TS within the BW (**b**); outside the BW, thus producing no relevant $I_{DS}$ variations (**c**); and within the BW while a compensation produces no relevant $I_{DS}$ variations (**d**). In the latter case, the presence of AFB1 reduces an existing TS peak (orange cross) and enhances another TS peak (orange peak). The effects of AFB1 are highlighted in orange.

In addition, we explained in Section 2.1 that the S to D transmission mainly occurs in the energy range referred to as BW, i.e., in between $E_{FD}$ and $E_{FS}$. Modifications of TEs and TS(E) can be included, partially included or not included in the BW, see Figure 4, leading to a voltage-dependent sensor response. Moreover, the current, $I_{DS}$, is directly proportional to the integral of TS, Equation (1). Thus, the modifications of TS(E) can compensate for each other in the BW in terms of $I_{DS}$ current. For example, Figure 4d shows the case where there is an increase in TS at a given energy (*E*) cancelled by a decrease in TS at another *E*. No significant $I_{DS}$ modulation occurs because of the compensation of the two effects.

Finally, $V_{GS}$ also alters the transmission function TS by shifting it toward higher/lower *E* according to the $V_{GS}$ polarity. As a secondary effect, $V_{GS}$ also affects the magnitude of TS (i.e., the transmittivity) because of the charging effect that can locally shift a TS peak or alter its transmittivity. Then, the presence of AFB1 may alter the new TS obtained for a specific $V_{GS}$ (and $V_{DS}$) in the same way. Therefore, we expect a voltage-dependent sensor response, both in terms of $V_{GS}$ and $V_{DS}$.

## 3. Methodology and Computational Methods

We investigated the chemical–physical and the electron transport properties of the MolFET SMS through ab initio atomistic calculations. We used the quantum chemistry package ORCA [69] to perform the isolated geometry optimizations of the sensing element 8PyDT and the target analyte AFB1. In particular, we used unrestricted Density Functional Theory (DFT) with the exchange-correlation functional Becke, 3-parameter, Lee–Yang–Parr (B3LYP) under the Generalized Gradient Approximation (GGA), and we considered the van der Waals (vdW) correction, DFT-D3. We used the polarized valence triple-ζ (def2-TZVP) basis set [70,71].

We addressed the study of the AFB1 adsorption onto the Au-8PyDT SMS in QuantumATK [54] through DFT with GGA, the Perdew–Burke–Ernzerhof (PBE) exchange-correlation functional and the polarized double-ζ (DZP) basis set for all elements except Au, for which we use the polarized single-ζ (SZP) basis set. We additionally used vdW Grimme DFT-D3 correction and the counterpoise (CP) correction for the Basis Set Superposition Error (BSSE).

**Figure 5:**
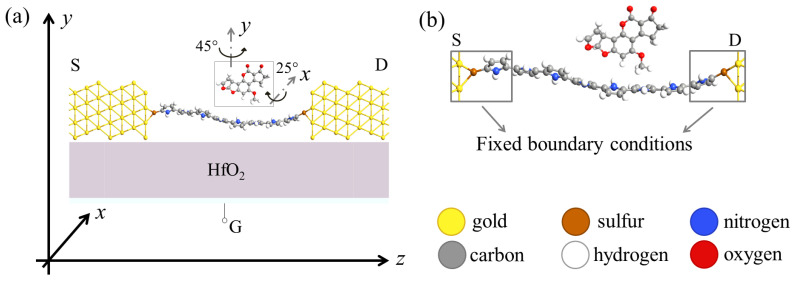
(**a**) Pictorial representation of the adsorption study strategy. (**b**) Example of a simulated adsorption geometry. The figure highlights the atoms which were kept fixed during the adsorption study to emulate fixed boundary conditions.

Compared with our previous work [26], this work extends the study to additional adsorption configurations to cover all possible realistic cases. Figure 5a reports the strategy used to study the adsorption of AFB1 to 8PyDT. In particular, starting from a random initial position of AFB1, we covered *y*-direction rotations with a rotational step of $45^{\circ }$ and *x*-direction rotations with a rotational resolution of $25^{\circ }$. The 8PyDT detection element repetition symmetry and hindrance make additional *x* rotations unnecessary. We placed the AFB1 in between a 2 Å and 2.65Å distance from the 8PyDT (that is naturally bent, leading to non-constant distance), and then we allowed the system to freely relax to achieve the most stable configuration, also accounting for *z*-direction rotations or adjustments. In all 40 adsorption configurations, we considered a reduced gold electrode structure to save computational time, and we accounted for the gold electrodes by exploiting fixed atom boundary conditions in the geometry optimization, as depicted in Figure 5b. Geometry optimization was performed for the AFB1 and the central portion of the Au-8PyDT. We used the built-in LBFGS method for the total energy minimization with a force tolerance of 0.05 eV/Å. The adsorption energy, $E_{ads}$, was therefore evaluated from its definition:

(5) $$E_{ads}=E_{AFB1/SMS}-\left[E_{AFB1}+E_{SMS}\right]$$

where $E_{AFB1/SMS}$ is the total energy of the AFB1 + Au-8PyDT system, $E_{AFB1}$ is the total energy of the isolated AFB1 and $E_{SMS}$ is the total energy of the isolated Au-8PyDT SMS.

We then chose the two most stable (i.e., probable) adsorption configurations and investigated the electronic structure and transport properties of the related Au-8PyDT MolFET SMS. We modeled a gate stack composed of a HfO_2_ layer and G electrode through a dielectric continuum region and a Perfect Electrical Conductor (PEC), respectively. Indeed, the gate stack does not affect the chemical structure and electronic structure properties of the molecular channel, whereas it determines the electrostatics of the system. The system was studied with QuantumATK using DFT GGA PBE vdW D3. A DZP basis set was used for all elements except for gold, which was described with SZP. We derived the system electrostatics by solving Poisson’s equation through the conjugate gradient method. Periodic boundary conditions were enforced in the *x*-direction to account for the extension of gold electrodes, and Dirichlet boundary conditions were enforced in the *z*-direction. In the *y*-direction, Dirichlet boundary conditions were applied to avoid periodicity artefacts caused by the presence of AFB1. Neumann boundary conditions at the gate electrode were assumed to model the gate metal correctly.

We used the NEGF formalism to calculate the electron transport as described in Section 2.1. Equations (1) and (3) estimate the current, $I_{DS}$. We specifically solved the electrostatic and transport equations self-consistently, with convergence achieved on the Hamiltonian, as a mixing variable, at a tolerance of $10^{-4}$.

Since, as addressed in Section 2.3, the SMS under study relies on an amperometric detection principle, we evaluated the sensor response to AFB1 as the $I_{DS}$ modulation caused by the AFB1 molecule. We calculated the absolute and relative $I_{DS}$ variations as:

(6) $$\begin{array}{cc} \; & \Delta I_{DS}=I_{DS,0}-I_{DS,AFB1}\\ \; & \Delta I_{DS\%}=\left[\left(I_{DS,0}-I_{DS,AFB1}\right)/I_{DS,0}\right]\;\cdot \;100 \end{array}$$

where $I_{DS,0}$ and $I_{DS,AFB1}$ are the current flowing in the isolated Au-8PyDT SMS and flowing in the SMS in the presence of AFB1. Note that a negative/positive $\Delta I_{DS}$ indicates an increase/decrease in current due to AFB1.

Finally, we studied the gating effect on the sensing performance of the Au-8PyDT SMS FET through a parametric electrical characterization. The initial guesses for the range of the gate voltage, $V_{GS}$, were chosen by inspecting the equilibrium transmission spectrum, TS(E) (i.e., null $V_{DS}$ and $V_{GS}$). Then, by supposing a possible gate coupling factor and a certain BW (i.e., $V_{DS}$), we qualitatively determined the range of $V_{GS}$ that permits the inclusion within the BW of useful molecular transmission states of all the considered configurations of Au-8PyDT SMS, with and without AFB1. The investigation aims to enhance the SMS response to AFB1. Hence, transmission states were considered useful when their transmittivity and broadening change significantly when AFB1 is in the vicinity of the 8PyDT channel. Thus, their introduction in the BW, triggered by the gate, alters the current, $I_{DS}$. According to Equation (1), such a TS difference affects the resulting current, $I_{DS}$, and thus also the current difference, $\Delta I_{DS}$, Equation (6). After the completion of the electrical characterization, the final value of $V_{GS}$ providing the largest TS(E) difference in the BW was chosen, and the resulting $\Delta I_{DS}$ and $\Delta I_{DS\%}$ were determined according to Equation (6).

## 4. Simulation Results and Analysis

### 4.1. Adsorption Configuration

Table 1 reports the adsorption energies of all 40 considered adsorption configurations. All the configurations with rotation in *y* equal to $0^{\circ }$, as well as the configurations with $y=90^{\circ }$ and $x=-45^{\circ }$ and $0^{\circ }$, show a positive adsorption energy ($E_{ads}$), thus they are unstable. The other configurations show similar adsorption energies and are stable. In particular, the two most stable configurations (i.e., showing the lowest $E_{ads}$) have a y-direction of $270^{\circ }$ and x-rotations equal to $x=-25^{\circ }$ and $x=-45^{\circ }$. Since the two configurations have the same y-rotation, we refer to them by indicating the *x* rotation angle: $x=-25^{\circ }$ and $x=-45^{\circ }$ for clarity. The two configurations will be briefly indicated as rotY$-25^{\circ }$ and rotY$-45^{\circ }$. rotY recalls that the two configurations have the same rotation on the y-axis, i.e., $270^{\circ }$.

For both the stable configurations, the $E_{ads}$ values are indicative of the creation of hydrogen bonds between the AFB1 and the 8PyDT detection element. Indeed, they are greater than weak physisorption values (typically 1÷30 kJ/mol) but lower than strong chemisorption values (typically > 100 kJ/mol), and they perfectly match the hydrogen bond range (1÷170 kJ/mol) [68]. The articles [72,73] experimentally proved that AFB1 can create hydrogen bonds with organic detection elements (DNA and aptamers) with binding energies similar to the ones obtained in this work. Therefore, we conclude this is also the case with the pyrrole-based detection element. Considering the chemical structure of 8PyDT, the hydrogen bonds probably originate between the AFB1 carbonyl or methoxy groups and the NH secondary amines of the 8-PyDT, which act as proton donors. The advantage of hydrogen bond creation is related to the chemical specificity of the AFB1–SMS interaction. Indeed, sensor selectivity is often related to the chemical affinity of the detection element with the analyte. Moreover, relying on the principle described in Section 2.3, giving a key role to orbital deformation, the stronger the chemical interaction, the greater the expected sensor response.

In the following, we consider the two most stable AFB1 adsorption configurations onto the SMS. Indeed, the second most stable configuration, which shows comparable total energy with the first, can also be achieved during the AFB1 adsorption onto the SMS because of random chemical competitors and processes. Therefore, from now on, we investigate both the rotY$-25^{\circ }$ and rotY$-45^{\circ }$ configurations to understand whether small angle variations between the AFB1 and the 8PyDT detection element can significantly affect the sensor performance.

### 4.2. Equilibrium TS, Fermi Level Position and Contacts

**Figure 6:**
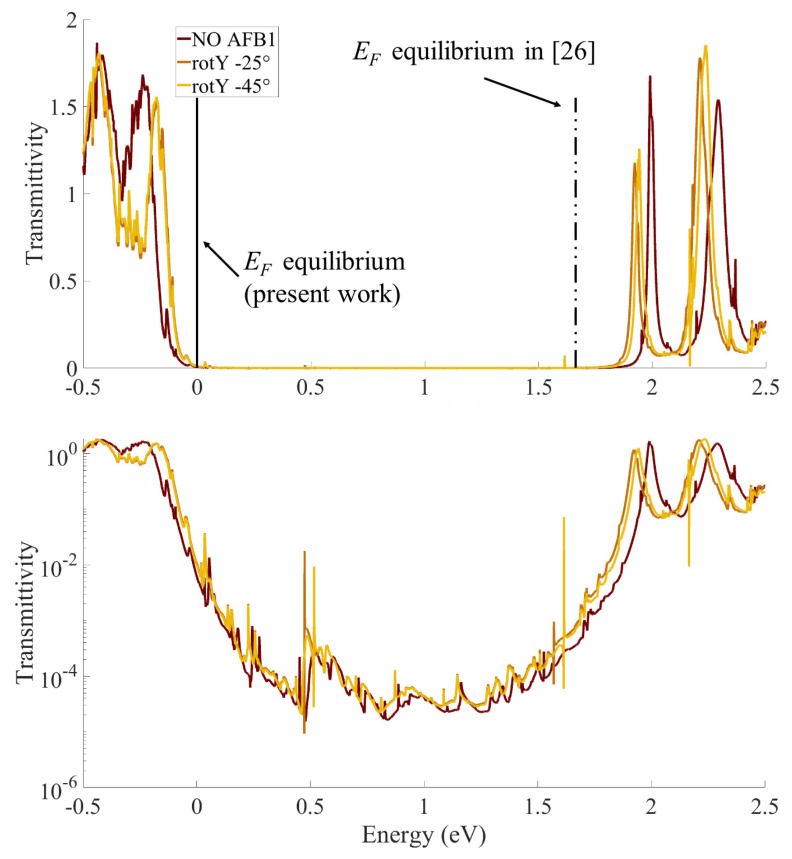
Computed equilibrium transmission spectra, TS(E), in the linear and semi-logarithmic scales, of an Au-8PyDT FET sensor with (dark red) and without the AFB1 target. The brown and yellow TS(E)s correspond to the two most stable configurations of the Au-8PyDT sensor in the presence of the target, i.e., an *x*-rotation of $-25^{\circ }$ and $-45^{\circ }$, respectively.

Figure 6 reports the equilibrium (i.e., $V_{DS}$ = 0 V and $V_{GS}$ = 0 V) TS(E) function for the SMS either without aflatoxin or in the presence of AFB1 in both rotY$-25^{\circ }$ and rotY$-45^{\circ }$ configurations. For clarity, the figure reports linear (top) and semi-logarithmic (bottom) scales. Note that the energy reference is chosen so that the system Fermi level, $E_{F}$, at equilibrium is 0 eV. Interestingly, a shift is present if comparing the obtained TS with our previous work [26]. In the present work, we find an $E_{F}$ close to the TS peak corresponding to the HOMO molecular level (low energy, left side), while in our previous work [26], $E_{F}$ was closer to the LUMO TS peak (high energy, right side). We relate this difference to the different S and D electrodes in the two works. The number of atoms and the orientation of the gold electrodes is the same in the two works (Face Centered Cubic (111) for both S and D). Nevertheless, in [26], we did not use the G electrode and we used periodic boundary conditions to model the electrode extension in all directions, including in the vertical direction. Instead, in the present work, we considered a gate electrode and we enforced Neumann and Dirichlet boundary conditions in the vertical direction to correctly model it. This corresponds to model broad and thin S and D electrodes, as it is the case when a gate is present in the structure (refer to Figure 1). Therefore, the considered S and D electrodes are very different from the ones in [26] and their Fermi levels are affected and shifted in energy. Consequently, when the equilibrium condition is reached through a charge (electron) redistribution in the system, the Fermi level alignment occurs at a different energy; the relative positions of the (broadened) HOMO and LUMO states of the molecular channel with respect to $E_{F}$ are varied. As a result, while in [26] the transport was mainly of LUMO-type, i.e., mediated by the LUMO TS peak, in the present study, the transport is naturally mediated by the HOMO TS peak. Nevertheless, as explained in Section 2.2, with a suitable $V_{GS}$ it is possible to shift TS in energy and recover the LUMO-mediated transmission.

The effect of AFB1 in both rotY $-25^{\circ }$ and rotY $-45^{\circ }$ configurations reduces the HOMO and LUMO TS peaks and shifts them, reducing the HOMO–LUMO Gap (HLG), i.e., the TS gap in between the HOMO and LUMO TS peaks. We relate the HLG reduction to the increased number of electron states (resulting from the increased number of atoms) between the S and D contacts when AFB1 is adsorbed. The broadening of the HOMO and LUMO TS peaks (width at half maximum) is instead the same in the two configurations. Indeed, the broadening functions depend on the material and geometry of the contact electrodes only, which are unchanged in the two configurations.

The two rotY $-25^{\circ }$ and rotY $-45^{\circ }$ configurations lead to very similar TSs, highlighting that a $20^{\circ }$ angle variation in the adsorption process does not significantly affect the transmission properties of the system. The semi-logarithmic scale highlights that the TS differences between the isolated SMS configuration and the ones with AFB1 are more marked in the LUMO peaks than in the HOMO ones. Therefore, the sensor response is expected to be larger for LUMO-mediated conduction than for HOMO-mediated conduction.

### 4.3. Two Terminal Device Current and Sensing

**Figure 7:**
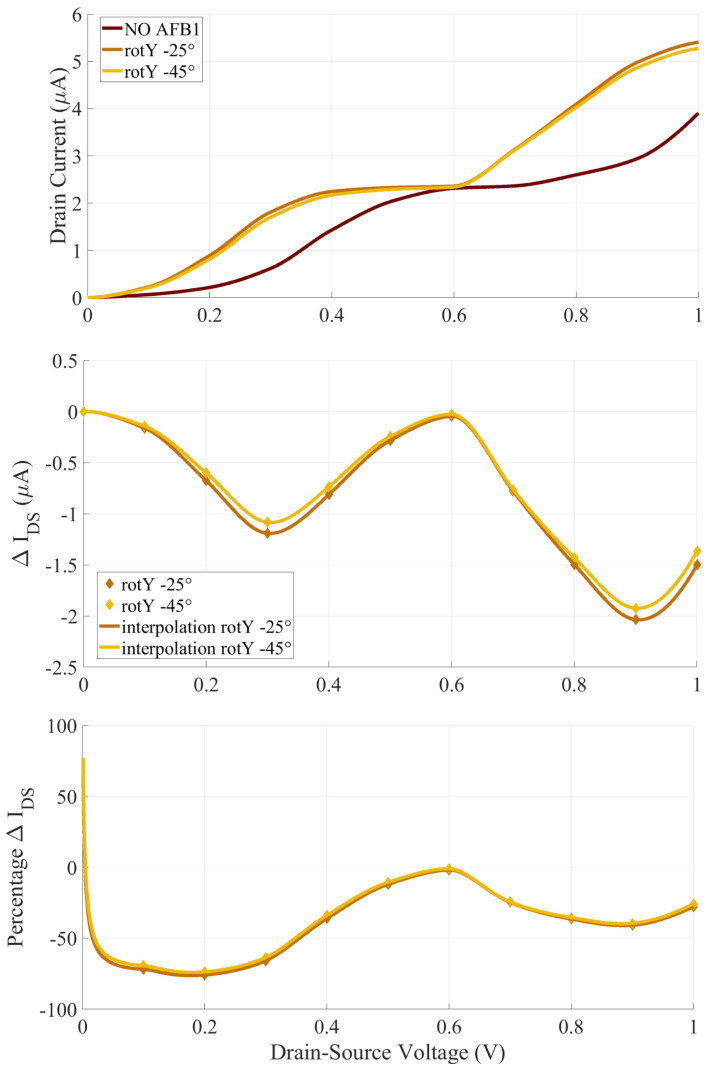
Computed current response of the Au-8PyDT sensor in the absence (dark red) and in the presence of the AFB1 target evaluated with null gating effect (i.e., $V_{GS}$ = 0 V).

Figure 7 reports the current–voltage $I_{DS}\left(V_{DS}\right)$ characteristics, the $\Delta I_{DS}$ and the $\Delta I_{DS\%}$ of the SMS with null G voltage ($V_{GS}$ = 0 V.) The reported current–voltage characteristics are compared with the cases with non-null $V_{GS}$, used to enhance the sensor’s performance in Section 4.4. The comparison allows to quantify the improved SMS sensitivity provided by the gating effect and thus justifies the use of an additional gate electrode in the Au-8PyDT SMS.

In both the rotY$-25^{\circ }$ and rotY$-45^{\circ }$ configurations, the AFB1 increases the $I_{DS}$ with respect to the isolated SMS one. In general, the dependency of TS on $V_{DS}$ should be considered for an appropriate evaluation of the current. In this specific case, it is possible to obtain a satisfactory explanation of the obtained trends by referring to the equilibrium TS in Figure 6. Indeed, since the Au-8PyDT junction is completely symmetrical on the S and D sides, the BW is symmetric with respect to the system $E_{F}$ (0 eV in Figure 6). When AFB1 is present, the HOMO peak moves closer to $E_{F}$ and the $V_{DS}$, permitting the HOMO TS peak to enter the BW, thus leading to a sharp increase in current up to $V_{DS}$ = 0.35 V. The $I_{DS}$ plateau between 0.35 V and 0.6 V can be explained by considering the sharp reduction in TS after the HOMO peak (left side, lower *E* in Figure 6), which does not significantly increase the Landauer integral of Equation (1). Then, for $V_{DS}>$ 0.6 V, the $I_{DS}$ increases thanks to the HOMO-1 and HOMO-2 peaks in the TS. Instead, without AFB1, the SMS HOMO peaks start conducting at higher $V_{DS}$ values (i.e., larger BW) and reach a plateau at higher $V_{DS}$ values. Furthermore, there is a smaller energy range with a low TS value between the HOMO and HOMO-1 TS peaks, and thus $I_{DS}$ starts increasing again after a smaller $V_{DS}$ value.

The $I_{DS}$ values obtained with the rotY$-25^{\circ }$ and rotY$-45^{\circ }$ configurations are very similar, showing again that a $20^{\circ }$ rotation in the adsorption configuration with respect to the expected one does not significantly affect the reliability of the amperometric detection. This is even clearer when the $\Delta I_{DS}$ ( Figure 7, middle) is considered. The current variation produced by the AFB1 is practically indistinguishable in the rotY$-25^{\circ }$ and rotY$-45^{\circ }$ configurations. Furthermore, two maximum (in absolute values) $\Delta I_{DS}$ are obtained at $V_{DS}$ = 0.3 V and $V_{DS}$ = 0.9 V, with a more than 1 μA and around 2 μA difference with respect to the SMS in the absence of AFB1. The obtained current variations are measurable with state-of-the-art amperometric techniques. Therefore, we identify the $V_{DS}$ values that maximize the $\Delta I_{DS}$ as possible biasing values for the two-terminal SMS case. The trend is confirmed by the $\Delta I_{DS\%}$ analysis. For a $V_{DS}$ between 0.1 V and 0.3 V, the $I_{DS}$ is doubled when AFB1 is present.

In the following, accounting for the great amount of noise that can affect such a nanoscale SMS performance, we will consider the gating effect to check if an advantage is achievable with the technological burden of an additional nanoscale G contact. We verify its usefulness in the following sections.

### 4.4. Gate Voltage Tuning and Gate Coupling Factor

Equation (1) states that the current is related to the TS through Landauer’s integral. Thus, a significant current variation caused by AFB1 is obtained if its presence modulates the TS within the BW. Starting with the knowledge of the most stable adsorption configurations from Section 4.1, we calculated the TS$(E,V_{DS}$ and $V_{GS})$ with AFB1 in the rotY$-25^{\circ }$ and rotY$-45^{\circ }$ configurations and we compared it with the TS obtained without AFB1. In general, TS depends on $V_{DS}$ and $V_{GS}$, which should be considered together. We chose the $V_{GS}$ that maximizes the differences in the TS(E) portion included in the BW in the case in which AFB1 is present with respect to the case without AFB1. The chosen optimal $V_{GS}$ was the gate voltage that maximizes the sensor current modulation and thus its sensitivity to AFB1.

**Figure 8:**
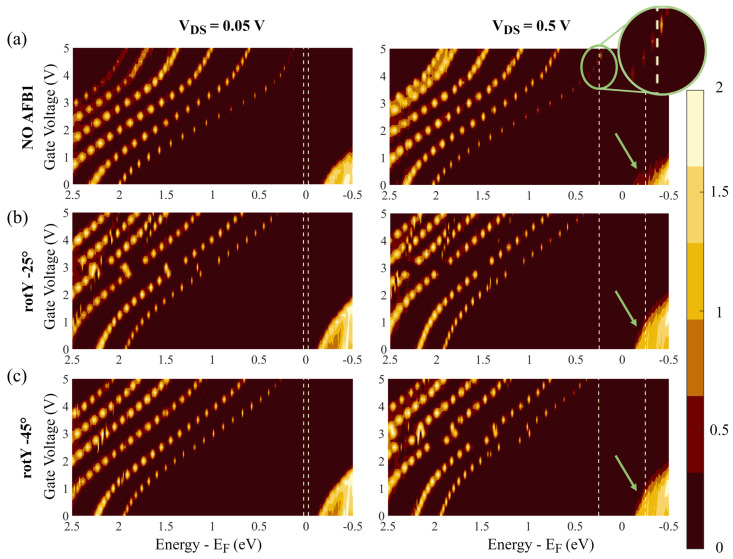
Computed contour diagrams of TS(E,= and $V_{GS})$ as a function of gate voltage, $V_{GS}$, and energy, *E*, for low (left side) and high (right side) values of $V_{DS}$ (0.05 V and 0.5 V, respectively). The pale yellow dashed lines denote the bias window, $-qV_{DS}$, whereas the green arrows indicate the HOMO transmission peaks entering the BW. (**a**) $TS(E,V_{GS})$ of the Au-8PyDT sensor in the absense of AFB1. The green inset is an enlargement of the contour diagram showing the LUMO peak entering the BW; (**b**) $TS(E,V_{GS})$ of the Au-8PyDT sensor in the presence of the target in the configuration rotY $-25^{\circ }$; (**c**) $TS(E,V_{GS})$ of the Au-8PyDT sensor in the presence of the target in the configuration rotY $-45^{\circ }$.

Figure 8 shows the contour diagrams of TS as a function of *E* and $V_{GS}$ for two fixed $V_{DS}$ values: a high $V_{DS}$ ( 0.5 V) and a low $V_{DS}$ ( 0.05 V) with and without AFB1. The abscissa axis (namely, the energy *E*) is reversed for graphical convenience. The HOMO and LUMO transmission peaks are on the right and left, respectively. The vertical dashed lines highlight the BW. For each of the considered configurations (i.e., isolated SMS, rotY$-25^{\circ }$ and rotY$-45^{\circ }$), the comparison between the low and high $V_{DS}$ graphs clarifies the existence of charging effects. If a charging effect is present, the transmission peaks should bend to oppose their entrance within the BW for large $V_{DS}$. Indeed, as energy levels get closer to the BW threshold, they should be filled/emptied by electrons. If a large charging energy is present, it presents a barrier to the filling/emptying process, thus revealing a resilience in contributing to conduction, see Section 2.1. In particular, considering that the analyzed cases are in the channel–electrode strong coupling regime, we expect a wide broadening and a small charging effect. Indeed, the reported cases show no significant slope variations between the low and high $V_{DS}$ cases. Thus, we exclude the charging effect from playing a major role in determining the transmission properties by varying $V_{DS}$ and $V_{GS}$ for the analyzed cases.

Considering the presence of AFB1, the HOMO TS peaks play a limited role. When AFB1 is present, the TS peaks at high and low $V_{DS}$ values behave almost identically, and they are included in the BW for high $V_{DS}$ only. The same occurs in the absence of AFB1 (green arrows in Figure 8). On the contrary, the calculation shows a more relevant role regarding the LUMO peak, which enters the BW at high $V_{DS}$ (top right) when AFB1 is not present for $V_{GS}=$ 4.75 V. See the green circle and relative enlargement in Figure 8. For the same $V_{GS}$ and $V_{DS}$, both the configurations with AFB1 (i.e., rotY$-25^{\circ }$ and rotY$-45^{\circ }$) show no LUMO TS peak in the BW. The mentioned phenomenon can be exploited to improve the sensor’s performance. We chose $V_{GS}=$ 4.75 V as the working point of the sensor since it allows LUMO-mediated conduction in all cases but with sensitivity with respect to AFB1. The presence of AFB1 reduces the LUMO TS peak height and brings it out of the BW, preventing it from contributing to the conduction. Instead, if AFB1 is absent, the LUMO TS enters the BW with a high transmission value (approximately 1.3), leading to an expected relevantly different current with respect to cases in which AFB1 is present.

From our analysis, the origin of the differences in the TS should be searched in the system parameters. We have already excluded the charging effect in the previous paragraphs. In both configurations with AFB1, the LUMO TS peaks start at an *E* slightly below 2 eV for null $V_{GS}$. As a result of $V_{GS}$, LUMOs shift toward lower energies, consistent with the description given in Section 2.2, but without reaching the BW threshold. Instead, the LUMO TS peak without AFB1 starts at exactly 2 eV. Similarly, with an applied positive $V_{GS}$, it is shifted toward lower *E*, yet it enters the BW at $V_{GS}=$ 4.75 V. Therefore, a larger LUMO TS shift in energy is obtained without AFB1 than the one obtained with AFB1 for the same change in $V_{GS}$. In other words, the AFB1 presence reduces the gate coupling factor, α, i.e., the effect of $V_{GS}$ on the energy level shift.

We suppose that the reduction in α caused by AFB1 presence is related to two main factors: (a) The AFB1 adds extra electron states in the molecular channel and this reduces the efficiency of the gate capacitance in controlling the channel charge. Indeed, for an unchanged $C_{G}$ value, the larger the number of states in the channel, the value of $V_{GS}$ which is necessary to induce the same percentage charge variation in the channel should be larger, intended as the number of molecular states with an electron population affected by the $V_{GS}$. This follows from the definition of capacitance as $C_{G}=\frac{\partial Q}{\partial V_{GS}}$, where *Q* is the total channel charge, that should now be interpreted in terms of electron population in the channel. (b) The electron states introduced by AFB1 are somehow reachable with difficulty from the electrons populating the SMS contacts. Indeed, AFB1 creates a hydrogen bond with the SMS, making electron sharing between the two structures difficult. As a result, $V_{GS}$ can vary the charge (i.e., electron orbital) in the channel thanks to the covalent bonds between the 8PyDT and contacts, while it barely influences the AFB1 charge (electron population), which is physically isolated from the rest of the SMS. Therefore, the $V_{GS}$ easily influences only the charge of the 8PyDT, which is only a portion of the channel, with a loss of efficiency in controlling the channel states and the channel charge, *Q*.

### 4.5. Sensor Response Enhancement through $V_{GS}$

**Figure 9:**
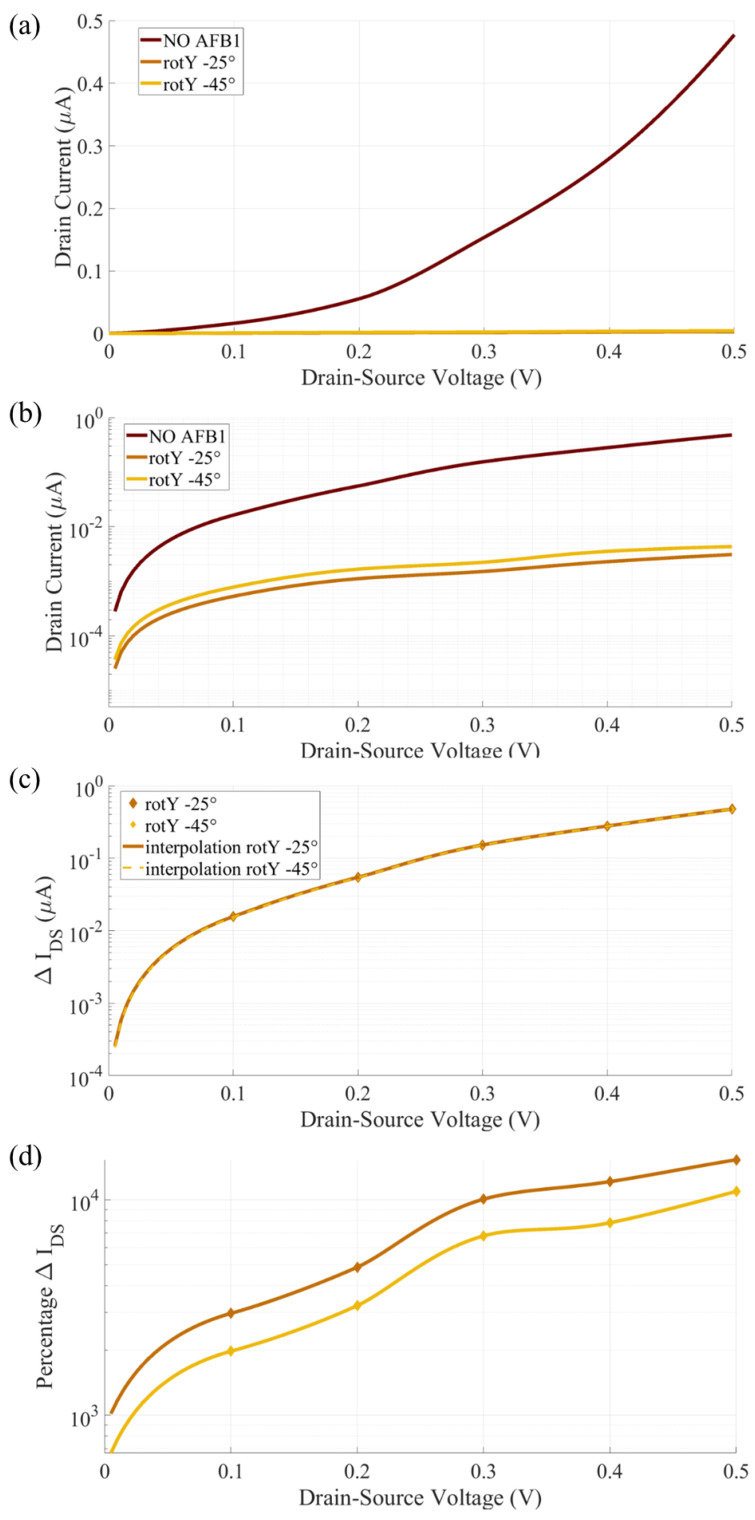
Computed current response in linear (**a**), semi-logarithmic scale (**b**), $\Delta I_{DS}$ (**c**) and $\Delta I_{DS\%}$ (**d**) of Au-8PyDT FET sensor for $V_{GS}$ = 4.75 V without (dark red) and with the AFB1 target (yellow and brown lines).

Figure 9 reports the $I_{DS}\left(V_{DS}\right)$ in linear (a) and semilogarithmic (b) scales and the $\Delta I_{DS}\left(V_{DS}\right)$ and $\Delta I_{DS\%}\left(V_{DS}\right)$ for a gate voltage fixed at $V_{GS}=$ 4.75 V. The performance improvement produced by the presence of the $V_{GS}$ is enormous. Indeed, in the $V_{DS}$ range, the $I_{DS}$ with AFB1 in both the rotY$-25^{\circ }$ and rotY$-45^{\circ }$ configurations is two orders of magnitude lower than the $I_{DS}$ obtained with the SMS without AFB1. The $I_{DS}$ values obtained without AFB1 are on the order of fraction of μA, making them detectable with conventional current-to-frequency converters or other well-established amperometric detection techniques. On the other hand, the $I_{DS}$ values obtained with AFB1 are on the order of nA, making them clearly distinguishable from the previous one and robust to noise. From the $\Delta I_{DS}\left(V_{DS}\right)$ analysis, Figure 9c, at low bias (e.g., $V_{DS}=$ 0.1 V) there is a reduction of around two orders of magnitude in $I_{DS}$. We identify $V_{DS}=$ 0.1 V as a possible working point for the amperometric detection of AFB1 through the studied SMS, with the advantage of having a large and also stable sensor response with operating $V_{DS}$ variations due to, e.g., supply noise or biasing circuit non-idealities. The percentage current variation, Figure 9d, is always above 1000%, and it overcomes 10,000% for a $V_{DS}$ close to 0.5 V.

### 4.6. Transmission Properties in Presence of AFB1

To understand the physical origin of the significant performance improvement obtained with the chosen operating gate voltage, we investigated the transmission properties of the SMS with and without AFB1 at an arbitrary $V_{DS}$ value of 0.5 V.

**Figure 10:**
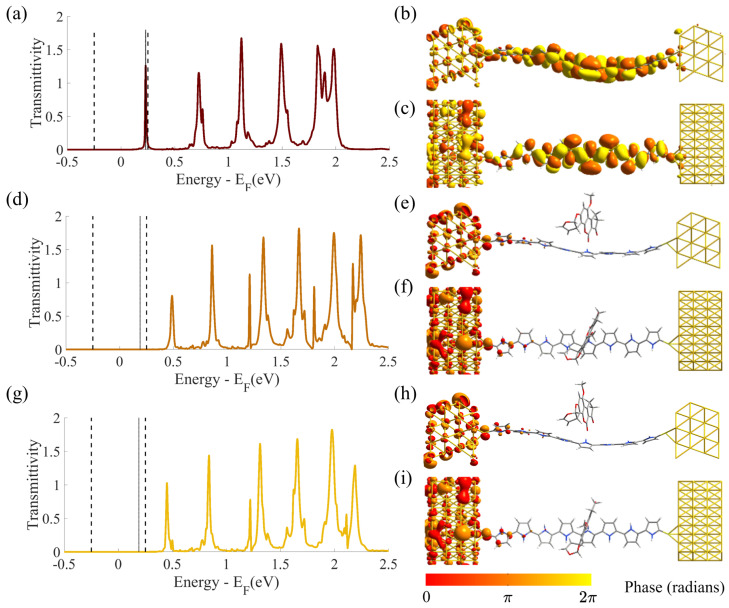
Computed transmission spectra TS(E) of the Au-8PyDT FET sensor for $V_{GS}$ = 4.75 V and $V_{DS}$ = 0.5 V without (**a**) and with the AFB1 target, with an *x*-rotation of 25${}^{\circ }$ (**d**) and 45${}^{\circ }$ (**g**). The dashed black lines represent the BW. The solid black lines show the energy values of the transmission peak included within the BW with the highest transmittivity (the transmission peaks in the cases with AFB1 are not visible on the linear scale). For these energy values (0.23 eV, 0.19 eV and 0.19 eV, respectively), the transmission eigenstates, TEs, with the highest transmission coefficient, TC, were computed and shown with the same isovalue (0.25) without AFB1 in front (**b**) and top (**c**) views (TC = 0.74), with AFB1 and *x*-rotation of 25${}^{\circ }$ in front (**e**) and top (**f**) views (TC = 0.0004) and with AFB1 and *x*-rotation of 45${}^{\circ }$ in front (**h**) and top (**i**) views (TC = 0.0005).

Figure 10 reports the TS(E) for $V_{GS}$ = 4.75 V and $V_{DS}$ = 0.5 V of the SMS and in the rotY$-25^{\circ }$ and rotY$-45^{\circ }$ configurations. The BW is highlighted by the vertical dashed lines. Furthermore, Figure 10 also reports the main TEs contributing, with TC, to the main TS peak within the considered BW. In the absence of AFB1, the TE is a large transmission isosurface delocalized over the entire 8PyDT detection element, connecting S and D with no obstacle to transmission, see Figure 10b,c.

Figure 10e,f,h,i reports the two TEs of the two largest TCs contributing to TS in the rotY$-25^{\circ }$ and rotY$-45^{\circ }$ configurations. Even if transmission isosurfaces are present on the left electrode (S electrode), the TE surfaces abruptly drop as one moves from the S to the 8PyDT. The transmission becomes negligible in the 8PyDT underneath the AFB1. By comparing the obtained results with the ones of the unperturbed 8PyDT, e.g., Figure 1, we further notice 8PyDT chain bending, which makes the NH group move closer to the AFB1 carbonyl (fifth pyrrole monomer from left). We associate the 8PyDT mechanical deformation with hydrogen bond formation. Indeed, the secondary amine NH acts as a proton donor and the oxygen of the AFB1 carbonyl termination thus attracts it. The mechanical deformation of the molecular channel spatially deforms the 8PyDT orbitals. Consequently, we expect a reduction or a break in the 8PyDT conjugation and electron delocalization, which in turn provokes an expected significant reduction in transmission and current. Indeed, the TEs are negligible in the proximity of AFB1. In addition, the TS LUMO peaks with AFB1 are at a higher energy with respect to the case without AFB1. We suppose the energy shift is related to mechanical torsion, which forces electrons to acquire higher angular momentum, increasing the orbital energy.

## 5. Conclusions

This work investigates the 8PyDT element used as a channel in a MolFET-based SMS for AFB1 single-molecule detection through ab initio atomistic simulations. With its intrinsic miniaturized size and steric hindrance repulsion, the 8PyDT molecule can detect a single AFB1 at a time and achieve the ultimate potential sensitivity at the single-molecule level. Our results demonstrate the AFB1 adsorbs into the Au-8PyDT molecular channel through a hydrogen bond. In addition, the presence of AFB1 produces a measurable current variation on the order of μA in the SMS. Furthermore, applying a suitable gate voltage of 4.75 V leads to a considerable amelioration of the sensing performance by increasing the SMS current modulation by two orders of magnitude when AFB1 is present. In particular, the AFB1 reduces the S to D transmission through a break in the 8PyDT conjugation caused by hydrogen bond formation, with a consequent TE, TS and $I_{DS}$ reduction from the μA scale to the nA scale. We also demonstrate that the current modulation is stable with small variations in the biasing $V_{DS}$ and with different rotations of AFB1 with respect to the 8PyDT detection element. Therefore, the analyzed sensor demonstrates robustness in the case of a non-ideal adsorption configuration.

Our results also demonstrate the possibility of exploiting a gate terminal to enhance the sensing performance of molecular quantum dots and to perform a direct electrical detection of single AFB1 molecules through the MolFET structure. The possibility to detect AFB1 by the direct and label-free measure of an electrical current paves the way for real-time and automatic monitoring of AFB1 in the field, in post-harvest storage and in manufacturing and production locations, with the additional advantage of avoiding complex testing protocols and the necessitation for skilled technicians to perform the measurements. The large current modulation possible with the gate voltage makes the sensor appealing for its potential robustness to external noise sources such as competitive chemical processes.

Finally, our work encourages future research efforts in the direction of miniaturized detection elements and MolFET for sensing purposes, motivating further investigation of the proposed sensor for AFB1 and other analytes, either on the modelling or experimental level.

## Figures and Tables

**Table 1:** Calculated $E_{ads}$ values.

*y* Rotation	*x* Rotation	$E_{\mathrm{ads}}$ (kJ/mol)	*y* Rotation	*x* Rotation	$E_{\mathrm{ads}}$ (kJ/mol)
$0^{\circ }$	$-45^{\circ }$	+159.26	$45^{\circ }$	$-45^{\circ }$	−54.29
	$-25^{\circ }$	+158.65		$-25^{\circ }$	−61.64
	$0^{\circ }$	+158.07		$0^{\circ }$	−57.57
	$+25^{\circ }$	+166.18		$+25^{\circ }$	−55.81
	$+45^{\circ }$	+152.81		$+45^{\circ }$	−58.68
$90^{\circ }$	$-45^{\circ }$	+161.23	$135^{\circ }$	$-45^{\circ }$	−49.19
	$-25^{\circ }$	−74.28		$-25^{\circ }$	−50.85
	$0^{\circ }$	+156.84		$0^{\circ }$	−49.96
	$+25^{\circ }$	−56.48		$+25^{\circ }$	−59.93
	$+45^{\circ }$	−67.66		$+45^{\circ }$	−60.61
$180^{\circ }$	$-45^{\circ }$	−45.60	$225^{\circ }$	$-45^{\circ }$	−53.64
	$-25^{\circ }$	−53.32		$-25^{\circ }$	−72.24
	$0^{\circ }$	−56.02		$0^{\circ }$	−82.07
	$+25^{\circ }$	−57.65		$+25^{\circ }$	−76.57
	$+45^{\circ }$	−63.07		$+45^{\circ }$	−78.54
$270^{\circ }$	$-45^{\circ }$	−86.75	$315^{\circ }$	$-45^{\circ }$	−70.95
	$-25^{\circ }$	−91.06		$-25^{\circ }$	−83.99
	$0^{\circ }$	−41.70		$0^{\circ }$	−54.41
	$+25^{\circ }$	−45.09		$+25^{\circ }$	−59.47
	$+45^{\circ }$	−53.19		$+45^{\circ }$	−52.94
